# Directed evolution to re-adapt a co-evolved network within an enzyme

**DOI:** 10.1016/j.jbiotec.2011.11.017

**Published:** 2012-01

**Authors:** John Strafford, Panwajee Payongsri, Edward G. Hibbert, Phattaraporn Morris, Sukhjeet S. Batth, David Steadman, Mark E.B. Smith, John M. Ward, Helen C. Hailes, Paul A. Dalby

**Affiliations:** a Advanced Centre for Biochemical Engineering, Department of Biochemical Engineering, University College London, Torrington Place, London WC1E 7JE, UK; b Department of Chemistry, University College London, 20 Gordon Street, London WC1H 0AJ, UK; c Department of Structural and Molecular Biology, University College London, Gower Street, London WC1E 6BT, UK

**Keywords:** GA, glycolaldehyde, HPA, hydroxypyruvate, PA, propionaldehyde, PP, pyrophosphate-binding domain, Pyr, pyrimidine-binding domain, SCA, statistical coupling analysis, TK, transketolase, TPP, thiamine pyrophosphate, Directed evolution, Coevolution, Transketolase, Enzyme engineering, Biocatalysis

## Abstract

We have previously used targeted active-site saturation mutagenesis to identify a number of transketolase single mutants that improved activity towards either glycolaldehyde (GA), or the non-natural substrate propionaldehyde (PA). Here, all attempts to recombine the singles into double mutants led to unexpected losses of specific activity towards both substrates. A typical trade-off occurred between soluble expression levels and specific activity for all single mutants, but many double mutants decreased both properties more severely suggesting a critical loss of protein stability or native folding. Statistical coupling analysis (SCA) of a large multiple sequence alignment revealed a network of nine co-evolved residues that affected all but one double mutant. Such networks maintain important functional properties such as activity, specificity, folding, stability, and solubility and may be rapidly disrupted by introducing one or more non-naturally occurring mutations. To identify variants of this network that would accept and improve upon our best D469 mutants for activity towards PA, we created a library of random single, double and triple mutants across seven of the co-evolved residues, combining our D469 variants with only naturally occurring mutations at the remaining sites. A triple mutant cluster at D469, E498 and R520 was found to behave synergistically for the specific activity towards PA. Protein expression was severely reduced by E498D and improved by R520Q, yet variants containing both mutations led to improved specific activity and enzyme expression, but with loss of solubility and the formation of inclusion bodies. D469S and R520Q combined synergistically to improve *k*_cat_ 20-fold for PA, more than for any previous transketolase mutant. R520Q also doubled the specific activity of the previously identified D469T to create our most active transketolase mutant to date. Our results show that recombining active-site mutants obtained by saturation mutagenesis can rapidly destabilise critical networks of co-evolved residues, whereas beneficial single mutants can be retained and improved upon by randomly recombining them with natural variants at other positions in the network.

## Highlights

► Previous single mutants of transketolase improved activity on new substrates. ► Recombination to form double mutants led to critical loss of functional expression. ► Mutated sites were found to be in a structural network of co-evolved residues. ► The network was re-adapted around previous mutants for kinetic synergy and functional expression.

## 1 Introduction

Targeted saturation mutagenesis of enzyme active-site residues can be used as an efficient alternative to error-prone PCR or DNA shuffling to engineer activity, specificity and enantioselectivity (Dalby, 2003; Park et al., 2005; Reetz et al., 2005; Paramesvaran et al., 2009; Bougioukou et al., 2009) as it targets the residues that most strongly influence catalysis and substrate binding. However, random point mutations selected only for activity often lead to a trade-off with protein stability (Beadle and Shoichet, 2002; Tokuriki et al., 2008) and their accumulation eventually leads to difficulties in introducing further mutations that improve function (Wang et al., 2002; Bloom et al., 2004, 2005).

The effects of random point mutations on protein stability or poor folding can be ameliorated by the addition of chaperones to allow the introduction of further activity enhancing mutations (Tokuriki and Tawfik, 2009). DNA shuffling can also be used to avoid the accumulation of destabilising mutations, as it tends to generate a greater proportion of functional variants with less trade off between activity and stability (Drummond et al., 2005). Alternatively, random mutagenesis to improve protein stability can be used to confer further evolvability for function (Bloom et al., 2006; Brown et al., 2010). For example, thermostability can be improved by consensus protein design approaches (Lehmann et al., 2002; Jackel et al., 2010), and also by the B-FIT method which targets saturation mutagenesis to residues with high crystallographic B-factors (Jochens et al., 2010; Reetz et al., 2010).

Some residues form co-evolved networks of synergistically coupled residues that can be identified by statistical coupling (or correlated mutation) analyses (Lockless and Ranganathan, 1999; Kuipers et al., 2009). Residues in such networks can be much more critical to protein stability and folding than the majority (Socolich et al., 2005), or confer other functional roles (Gobel et al., 1994; Halabi et al., 2009), including allostery (Suel et al., 2003), mediation of conformational changes (Nascimento et al., 2008), catalysis (Estabrook et al., 2005) and substrate specificity (Kuipers et al., 2009).

Correlated mutation analyses of enzyme families can be used to guide directed evolution to residues that influence substrate specificity (Kuipers et al., 2009). Conversely, networks of structurally coupled residues that improve thermostability have even been created by iterative saturation mutagenesis (Reetz et al., 2009), and synergy has also been obtained by analysing the results of the first rounds of random mutagenesis and recombining those predicted to be mutually beneficial (Fox et al., 2007). However, inadvertently disrupting such networks may lead to significant loss of stability or function and therefore represent a potential barrier to recombining and accumulating beneficial mutations during directed evolution, as we have found here for the transketolase enzyme.

Transketolase (TK) catalyses the enantioselective synthesis of α,α′-dihydroxy ketones from donors d-xylulose-5-phosphate or *β*-hydroxypyruvate (HPA), and acceptors d-ribose-5-phosphate, d-erythrose-4-phosphate or other aldehydes (Sprenger et al., 1995). HPA renders the reaction irreversible making it useful for industrial biocatalytic syntheses (Demuynck et al., 1991; Hobbs et al., 1993; Morris et al., 1996; Schenk et al., 1998; Turner, 2000; Shaeri et al., 2006; Ingram et al., 2007; Smith et al., 2010). Saturation mutagenesis was previously targeted independently to 10 active-site residues in contact with cofactor or substrate, and also to the 10 least conserved second shell residues (Hibbert et al., 2007), guided by TK structures (Littlechild et al., 1995; Nilsson et al., 1997), and an analysis of thiamine pyrophosphate (TPP)-dependent enzyme phylogeny (Costelloe et al., 2008). Various single mutants gave improved activity towards glycolaldehyde (GA) (Hibbert et al., 2007), or accepted propionaldehyde (PA) better with either enhanced or reversed enantioselectivity (Hibbert et al., 2008; Smith et al., 2008; Hailes et al., 2010). Several D469 mutations also accepted a range of linear aliphatic (Cazares et al., 2010) and aromatic (Galman et al., 2010) aldehydes. Chemical denaturation, dimer interface mutations and biocatalytic process conditions that destabilise *Escherichia coli* TK have also been well characterised (Brocklebank et al., 1999; Martinez-Torres et al., 2007; Aucamp et al., 2008).

Here, we show that the creation of double mutants from our previous activity-enhancing single mutants within the active site of *E. coli* transketolase, leads to loss of enzyme function. Activities and soluble expression levels of protein trade-off smoothly in the single mutants, whereas many of the double mutants disrupt both properties more extensively, suggesting that they fall below a critical threshold in stability or folding that then manifests as a loss of soluble protein expression and/or inclusion body formation, as observed previously (Calloni et al., 2005). Statistical coupling analysis (SCA) (Gobel et al., 1994; Lockless and Ranganathan, 1999; Suel et al., 2003) of a multiple sequence alignment for 382 related TPP-dependent enzymes revealed that 10 of the 11 active-site double mutants alter at least one of nine residues that form a single co-evolved network.

To test whether naturally occurring variants within this network could improve activity towards PA, or enhance our existing non-naturally occurring D469 mutants, we created a small library of single, double and triple mutants spanning seven of the co-evolved residues. Only naturally occurring mutations were allowed at each site, except D469 for which only variants previously found to improve activity towards PA were used. The use of only natural variants in so-called smart libraries has been shown recently to be an efficient way of improving enzyme activity and enantioselectivity (Jochens and Bornscheuer, 2010). A triple mutant cluster at D469, E498 and R520 was found to behave synergistically and a double mutant D469S/R520Q was obtained with significantly enhanced *k*_cat_, useful for biocatalytic applications. The R520Q mutation was also found to double the specific activity of our previously identified mutant D469T to create our most active TK variant to date.

## 2 Materials and methods

All chemicals were obtained from Sigma and used as supplied, except *β*-hydroxypyruvate (HPA) which was prepared as the lithium salt by modification of a previously described procedure (Morris et al., 1996).

### 2.1 Statistical coupling analysis of TPP-dependent enzyme protein sequences

The alignment of 382 sequences of the homologous PP and Pyr domains from 17 TPP-dependent enzyme types including transketolase, was described previously (Costelloe et al., 2008). The statistical coupling analysis (SCA) was carried out as described previously using the SCA Matlab toolkit (Version 1.5) with default parameters (Suel et al., 2003). Perturbations were allowed that resulted in sub-alignments greater than 0.21 as a fraction of the total alignment size. Following clustering, the statistical-coupling matrix was iteratively focused on areas of high signal and re-clustered. Further details are given in Supplemental Experimental Procedures.

### 2.2 Double mutant and library construction

All defined mutants and libraries were introduced into the tktA gene in plasmid pQR791 (includes a His tag), using the Quikchange method as described previously (Hibbert et al., 2007), then transformed into XL10-gold cells (Stratagene, La Jolla, CA) for expression of the TK mutants. All defined mutants were confirmed by DNA sequencing. Defined double and triple mutants were constructed from existing single mutant plasmids as templates and then step-wise mutagenesis using primers with optimal mutagenic codons. Seven of the nine cluster residues identified by SCA above were subjected to partial random mutagenesis (Table 1). Pro493 and Pro486 were excluded. Based on the alignment of the 382 TPP-dependent enzyme sequences, the four most common naturally occurring residues at six of the seven sites, and the previously identified S, A, L and T variants of D469, were each introduced individually into the wild-type plasmid. All primer sequences are given in Table S1 (available online). The 467/495 double-mutant library was constructed using an equimolar mixture of the four G467 mutant plasmids as template and an equimolar mixture of the four D495 mutagenic primers. The converse reaction was also performed with the D495 plasmids and G467 mutagenic primers, and the products combined (1:1) prior to transformation. A similar strategy was used to create the triple mutant library of D469/E498/R520 and each of the double mutant libraries D469/E498, E498/R520 and D469/R520. Colonies from each library were picked and cultured in individual wells of 96-deep-square-well plates, then divided into 96-well reaction plates as described previously (Hibbert et al., 2008). The total library contained 28 confirmed single mutants in triplicate, 90 random colonies from each of the four double-mutant libraries, and 540 random triple-mutant colonies. Each plate contained three wild-type colonies and three blank wells for internal reference. DNA sequencing of 20 random colonies identified 7 unique double mutants (out of 10), 7 unique triple mutant colonies (out of 10) and no wild-types. The remainder were three failed sequences and three duplications.

### 2.3 Colorimetric screening of libraries for activity

Colorimetric screening of TK mutants was carried out using reaction plates containing 100 μl of cell culture per well, lysed by a double freeze–thaw from −80 °C. The assay was carried out twice for each plate, for the reaction between 50 mM LiHPA and 50 mM PA in 50 mM Tris–HCl, pH 7.0 in the presence of Mg^2+^ and TPP cofactors at 21 °C for 1 h (Hibbert et al., 2008), using a tetrazolium-based colorimetric assay as described previously (Smith et al., 2006a), and absorbance measured with a plate reader (Fluostar, BMG-labtech).

### 2.4 Specific activities of selected mutants

The specific activities of selected TK mutants were obtained for reactions of 50 mM PA with 50 mM LiHPA, in 2.5 mM TPP, 9 mM MgCl_2_, 50 mM Tris–HCl, pH 7.0. Total activities were first obtained from clarified sonicated cell lysates using HPLC as described previously (Hibbert et al., 2008). TK concentrations were determined by SDS-PAGE densitometry as previously described (Hibbert et al., 2007) for total TK before clarification, and soluble TK after clarification. Soluble fraction concentrations were used in all specific activity calculations. A baseline reaction and expression level, from an XL-10 *E. coli* lysate in which exogenous transketolase is not overexpressed, was subtracted from all enzyme reactions, and expression level determinations. Predicted activities for the effects of combining multiple mutations was based on the additivity of free energy, and so determined by multiplying each relative enhancement of the WT activity.

### 2.5 Kinetics of purified enzymes

Wild-type and mutant TKs were over-expressed and purified using His-tag affinity chromatography, and enzyme kinetics determined as described previously (Hibbert et al., 2008). Reactions were carried out in triplicate at 0.027–0.26 mg/ml enzyme, 50 mM LiHPA, 0–75 mM PA, 50 mM Tris–HCl, 2.4 mM TPP, 9 mM MgCl_2_, 250 mM NaCl, pH 7.0, at 21 °C in sealed glass vials. Reactions were monitored every 2 h for 12 h using high resolution C18 HPLC as above after quenching 1:9 in 0.1% TFA, with detection of product by UV at 200 nm. HPLC was calibrated against the product standard obtained using our previously reported TK mimetic synthesis (Smith et al., 2006b).

### 2.6 Enantioselectivities obtained with purified enzymes

The stereoselectivities of purified TK wild-type and variants D469S, R520Q, D469S/R520Q and D469T were established by derivatisation and gas chromatography as described previously (Smith et al., 2008). Reactions were carried out to completion (48 h at 21 °C) using purified TK with 50 mM LiHPA, 50 mM PA, 50 mM Tris–HCl, 2.4 mM TPP, 9 mM MgCl_2_, 250 mM NaCl, pH 7.0 at 300 μl scale in sealed glass vials. Reaction mixture (100 μl) was transferred to vials containing 300 μl EtOAc, shaken and allowed to partition. The organic phase (100 μl) was transferred to fresh vials to which 20 μl pyridine, containing 10 mg/ml DMAP, was added. Following conversion of 1,3-dihydroxypentan-2-one (DHP) to the diacetate, enantiomeric purity was assessed by gas chromatography (15 psi He carrier gas) on a Perkin-Elmer Autosystem XL Gas chromatograph with a β-Dex 225 chiral column (Supelco, 30 m × 0.25 mm), 1 μl samples injected at 250 °C, a column temperature of 60 °C increased at 3 °C/min, and detection at 300 °C with a flame ionised detector (FID). Retention times obtained were: (3*R*)-pentan-2-one diacetate, 29.9 min; (3*S*)-pentan-2-one diacetate, 30.3 min.

### 2.7 Enantioselectivities obtained with enzymes from cell free lysates

For mutants D469Y, D469T/R520Q, D469Y/R520V and D469Y/R520Q e.e.s were determined using the following procedure modified from Cazares et al. (2010). MgCl_2_ (390 μl of 100 mg/ml solution, 0.4 mmol) was added to TPP (22 mg, 48 mmol) in water (10 ml) at pH 7. TK variant cell-free lysate (2 ml) was added and the mixture incubated at room temperature for 20 min. In a separate flask, Li-HPA (110 mg, 1.0 mmol) was dissolved in water (10 ml) and the pH adjusted to 7. This was then added to the enzyme suspension with PA (72 μl, 1.0 mmol) and stirred at rt for 48 h. During this time, the pH was maintained at 7.0 by addition of 1 M HCl using a pH stat (Stat Titrino, Metrohm). Silica was added and the reaction mixture concentrated to dryness, dry loaded onto a flash silica gel column, and purified using flash silica chromatography (EtOAc:hexane, 3:7) to give DHP as a colourless oil, (*R*_f_ = 0.20, EtOAc:hexane, 1:1). DHP was monobenzoylated at the primary alcohol for chiral HPLC analysis. To a stirred solution of 1,3-dihydroxypentan-2-one (13 mg, 0.11 mmol) in CH_2_Cl_2_ (5 ml) was added 2,4,6-collidine (14 μl, 0.11 mmol) and benzoyl chloride (21 μl, 0.11 mmol). The reaction was stirred overnight, then washed with HCl (2 M, 25 ml) and the product extracted with CH_2_Cl_2_ (20 ml). Silica was added and the reaction mixture concentrated to dryness, dry loaded onto a flash silica gel column, and purified by flash silica chromatography (EtOAc:hexane, 1:9). Solvent was removed under reduced pressure to give the monobenzoylated ketodiol as a colourless oil (22 mg, 91%) (*R*_f_ = 0.17, EtOAc:hexane, 2:8). The monobenzoylated product was then dissolved in hexane:2-propanol (1:1) to a final concentration of 1 mg/ml. Chiral HPLC analysis was performed on a Varian Prostar instrument equipped with a Chiracel AD chiral column (Daicel; Chiral Technologies Europe, France) 25 cm × 0.46 cm and hexane:2-propanol (95:5, 0.8 ml/min) gave retention times of 22.0 min (*R*-isomer) and 24.5 min (*S*-isomer).

## 3 Results and discussion

### 3.1 Recombination of known single mutants

The recombination of previously identified single mutants led to an unexpected loss of function. The TK mutations A29E, H461S and R520V, found previously to improve activity towards both glycolaldehyde (GA) and propionaldehyde (PA) (Hibbert et al., 2007, 2008), were recombined by site directed mutagenesis to all possible double and triple mutants. D469T and D469A, also found previously to improve activity towards PA only, were similarly recombined with D259A, D259G, D259S and D259Y single mutants known to improve activity towards both PA and GA (Hibbert et al., 2007, 2008). The double mutants were expressed and clarified as sonicated lysates, and used to determine their total soluble TK expression levels, and also their specific activities for the formation of both erythrulose from GA, and 1,3-dihydroxypentan-2-one (DHP) from PA. In every case, the specific activities of the double mutants were lower than their respective single mutants on both substrates, and achieved only a fraction of the activities expected if the effects of the two mutations were simply additive (see Figure S1 available online). Interestingly, the ratio of specific activities for PA/GA increased considerably for D469T/D259S (0.66), D469T/D259Y (0.55), D469A/D259S (0.58), D469A/D259Y (0.54), compared to wild type (0.04), and the single mutants D469T (0.38), D469A (0.20) and D259A/G/S/Y (all 0.03–0.06). However, the overall loss of specific activity towards both substrates diminished the biocatalytic usefulness of these double mutants.

**Fig. 1:**
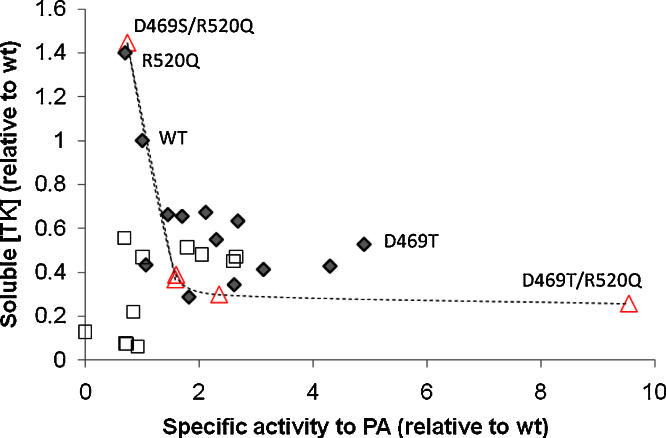
Trade-off between soluble TK expression and the specific activity towards propionaldehyde for a series of previously identified single mutants (), and the initially created double mutants obtained by recombining the singles (□). Double and triple mutants obtained from the new library guided by SCA, and also for D469T/R520Q, are shown as triangles (▵) and interpolated with a dashed line.

The expression levels for soluble TK were measured in the clarified sonicates for all mutants (see Figure S2), and for the single mutants these were found to trade off smoothly against their specific activities (Fig. 1). This was likely to have resulted from partial loss of protein stability as expected when accumulating mutations for activity by random mutagenesis (Bloom et al., 2006), and whereby loss of stability can lead to loss of soluble expression in the cell (Calloni et al., 2005). Consistent with this link between stability and soluble expression, D469T gave 53% of the soluble expression level observed for wild type, and a decrease in the temperature at which aggregation was induced from 58 °C for wild type (Jahromi et al., 2011), to only 47 °C for D469T (Supplemental Experimental Procedures & Figure S3). By contrast, most of the double mutants were found to lose both activity and soluble expression levels much more markedly than the single mutants, suggesting that the loss of protein stability had crossed a critical threshold beyond which little of the protein was functionally folded. Such a significant loss from just two mutations in this large enzyme was unexpected and hinted at a strong synergy between the pairs of residues mutated. Given that the number of residues in coevolved networks that are critical to stability and folding is often relatively small (Socolich et al., 2005), and that reciprocal sign epistasis appears to be relatively rare in protein evolutionary pathways (Carneiro and Hartl, 2010), loss of stability from accumulating point mutations is normally expected to be gradual and smooth, until the free energy no longer favours the native fold (Bloom and Arnold, 2009). Indeed this appears to be the case for our single mutants. However, synergistic networks might be expected more frequently in functionally important regions that are more conserved, such as an enzyme active site, to retain a correctly folded pocket, cofactor binding, and efficient catalysis. Saturation mutagenesis targeted to single residues of the active site is therefore potentially disruptive to existing synergistic networks of residues, yet also capable of improving function while retaining a just sufficient level of protein folding or stability. Simple recombination of two similarly identified mutations at the threshold of folding or stability would therefore be expected, as observed, to lead to complete loss of function in nearly all double mutants. This prompted us to use statistical coupling analysis (SCA) (Lockless and Ranganathan, 1999; Suel et al., 2003) to identify coevolved sites that may have become disrupted by our single and double mutants.

### 3.2 Statistical coupling analysis

**Fig. 2:**
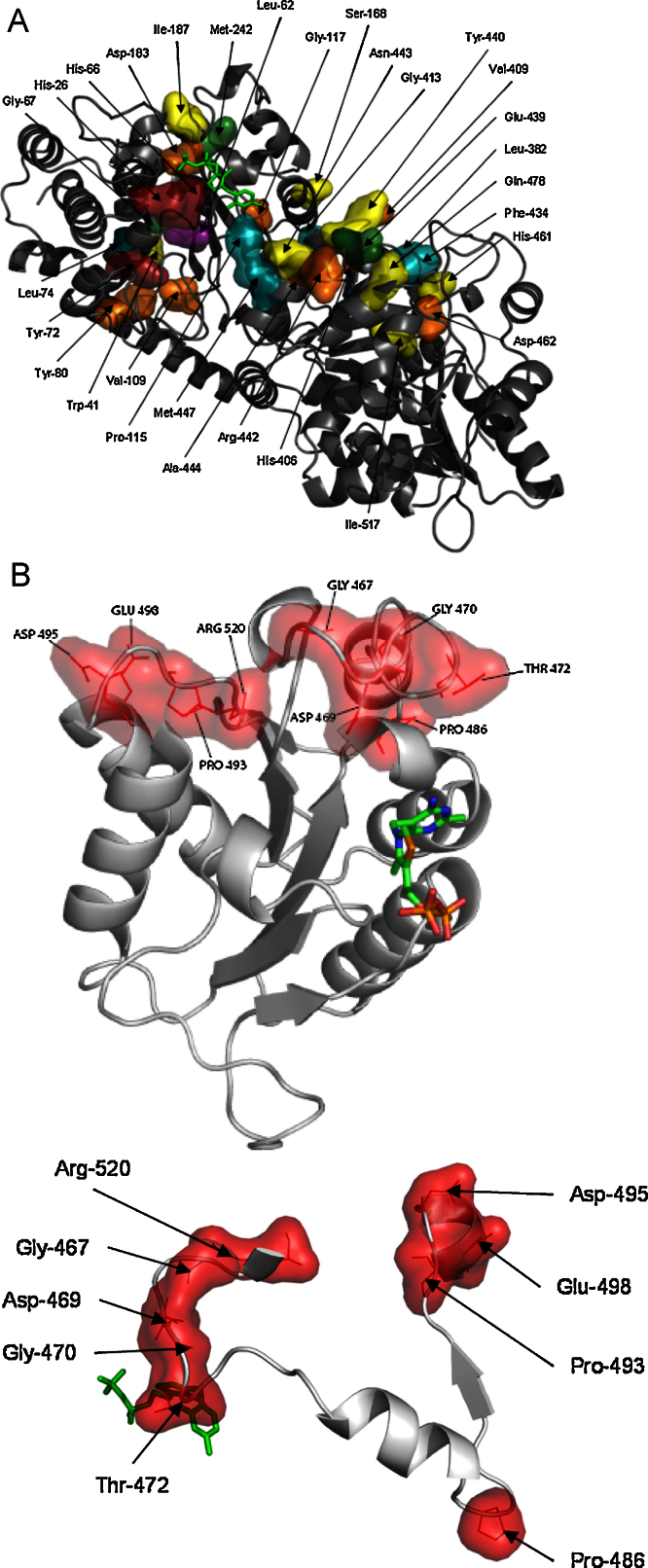
Statistical coupling analysis (SCA) of the PP- and Pyr-domains of *E. coli* transketolase. (A) One monomer of the TK homodimer is shown as grey ribbons. Six clusters (each coloured differently), form a single large network at the dimer interface, as revealed by SCA of the combined PP–Pyr domains. (B) Nine-residues form a single co-evolved network (red surface) within the Pyr-domain. The top view shows the network within the whole Pyr domain (grey ribbons). The bottom view is rotated for a clearer view of only the networked residues (red surface) and interconnecting sequence (grey ribbons). The TPP cofactor is shown in sticks. Images were produced with PyMOL (http://www.pymol.org) and the *E. coli* TK structure 1QGD.pdb (Littlechild et al., 1995). (For interpretation of the references to colour in this figure legend, the reader is referred to the web version of the article.)

A previously constructed alignment of 382 TPP-dependent enzyme sequences (Costelloe et al., 2008) provided a diverse but evolutionarily related basis set to perform the SCA. The analysis was applied to the PP and Pyr domains in tandem and also individually for each domain (matrices of statistical coupling energies and corresponding dendrograms are shown in Figures S8 and S9). The C-terminal domain was omitted as this does not form part of the two identical enzyme active sites, although a potential role in enzyme regulation has been previously postulated (Nikkola et al., 1994). Analysis of the combined PP–Pyr domain sequences revealed several strongly co-evolved clusters of between three and nine residues. These clusters interconnected to form a larger co-evolved network predominantly at or underpinning the dimer interface between the PP and Pyr domains (Fig. 2A). One of the clusters H26/H66/Y72, contained two histidines that can interact with substrate in the active-site. Another contained one of the mutated residues, namely H461 normally associated with the binding of phosphate moieties in natural substrates.

Independent analyses of the PP and Pyr domains were carried out to suppress the statistical signal from the dimer interface residues, and reveal any co-evolved networks entirely within each domain. SCA of the PP domain revealed only the H26/H66/Y72 cluster, and a nine residue cluster (comprising W41, L74, G67, L62, I187, P115, G117, S168 and M242), both present in the network from the combined PP–Pyr analysis (data not shown). However, analysis of the Pyr domain alone resolved a new network of nine residues that displayed similar statistical variance upon perturbation (Figure S9B), and included two of the previously mutated residues D469 and R520. This cluster in the Pyr domain was found also to group within the protein structure which further validates the analysis. Visualisation of the identified nine positions showed that they form two major spatial clusters such that P493, D495, and E498 cap the N-terminus of an alpha helix (Fig. 2B). This helix is 9 Å from R520 in the second group, which packs against an active site loop comprising G467, D469, G470 and T472. Finally, residue P486 forms the basis of a hairpin turn that brings the helix and loop clusters close together in space, but does not interact directly with either of the two main clusters, and is 10.5 Å from the closest coupled residue T472. Removal of the structurally important P486 from the analysis also removed P493, the closest residue to P486 in the sequence.

D469 forms several hydrogen bond interactions, particularly with the highly conserved residues H26 and R91 within the other monomer, and therefore has the potential to be structurally disruptive upon mutation. It also forms an electrostatic interaction over 7 Å with the positive charge on the R520 sidechain, which may explain their presence in the same co-evolved cluster. In addition, R520 forms a salt bridge to E468 which is adjacent to G467 and D469 within the co-evolved cluster. E468 is highly conserved and was therefore unlikely to be resolved by SCA, yet it is highly likely on structural considerations to mediate couplings within the identified co-evolved cluster. D469 also forms a similar electrostatic interaction over 6.4 Å with the positive charge on the H461 sidechain.

A29 and D259 were the only mutated residues in our initial recombination study that were not present in any cluster. Every double mutant constructed had therefore altered at least one residue (H461, D469 or R520), from a co-evolved network, with all but one affecting the nine residue network contained within the Pyr domain. In every case, the networked residues were also always mutated to amino acids that were not present in natural sequences at that position. While D259 was not found in a network, it cannot be fully ruled out of such a role as it has no equivalent in most TPP-dependent enzymes. The significantly reduced sequence set at this position may therefore have led to a relatively low signal in the SCA.

D259 is in a surface helical loop which forms part of the active site entrance, and forms critical hydrogen bond interactions to the T256 and H258 sidechains. Mutation of D259 to A, G, S or Y could easily therefore lead to destabilising structural disruption of one or more loops around the active site. A29 is an active-site residue that becomes buried within a cavity upon TPP cofactor binding, such that the methyl side chain comes into direct contact with the terminal phosphate of TPP. Mutation of this residue can therefore potentially affect the binding and stability of the TPP cofactor and the associated stability of the holo-enzyme. The inability to recombine the single mutants in every case may therefore stem from the addition of a second destabilising mutation to one that already significantly disrupts a critical network. In several cases, both mutations were in either the same or two different networks and therefore expected to be particularly disruptive.

### 3.3 Mutagenesis and screening for propionaldehyde activity

Given that the non-natural variations are selected against at co-evolved network residues in natural sequences, but that the single mutants were not fully disruptive, it was anticipated that at least one beneficial single mutation might be retained while exploring alternative natural variations at the coupled sites to improve either the soluble expression level or the activity to PA. Most of the best single mutants were at residue D469 and so this was chosen as the set of non-natural single variants to retain in a new library. The two proline residues may also result in significant disruption to the protein structure, particularly P486 which ensures that the two clusters in the Pyr-domain are brought into proximity. Therefore, only the seven Pyr-domain cluster sites G467, D469, G470, T472, D495, E498 and R520 were targeted for both site directed and partial saturation mutagenesis.

Our aim was to select for high activity towards the PA substrate that built upon and therefore retained the previously identified mutants of D469 (S, A, L and T), in the new libraries, and using only natural variants at the remaining six sites in an attempt to complement the non-natural D469 mutations. The other six sites were initially subjected to site-directed mutagenesis of the wild-type sequence, to independently obtain single mutants of only the top four most frequently occurring natural variations at each position, as determined from the alignment of the 382 TPP-dependent enzyme sequences (Table 1). These were then assayed for total activity towards both 50 mM GA and 50 mM PA substrates. None of the mutants at D495 and E498 gave activities above 70% that of the wild-type enzyme. However, all other residues gave at least one mutant with a total activity greater than that for wild type.

The network is formed mainly from two spatial clusters (Fig. 2B) with one containing D469, and the other comprised entirely of D495, E498 and P493, from which neither of the D495 or E498 libraries produced any variants with improved PA activity. Four double and one triple mutant libraries (Table 1), were constructed to investigate potential synergies between residue pairs within the same spatial cluster, and between the two main spatial clusters, with a particular focus on retaining D469 mutants. The D469/R520 library was chosen as both sites produced single mutants with improved PA activity, and the two sites are structurally very close within one of the major spatial clusters. The D469/E498 library explored the potential for synergy between the two major spatial clusters, in which one of the sites retained D469 variants, while the other gave single mutants less active than wild type. The G467/D495 library was chosen as G467 gave at least two mutants with potentially similar activity to those of D469, and also explored a potential synergy to D495 in the other spatial cluster. Finally, the E498/R520 library was chosen to explore the third pairing within the triple mutant library D469/E498/R520.

Double mutant libraries that produced variants with activities of at least 70% relative to wild type included G467/D495, D469/R520, and E498/R520. Of these, only D469/R520 produced variants that were more active than the wild-type enzyme, including D469S/R520Q. By contrast, D469/E498 did not produce any variants with at least 70% wild-type activity. However, the triple mutant library yielded two variants D469S/E498D/R520Q and D469S/E498D/R520A with activity greater than wild type. Therefore, single and double mutants containing variants at E498 were generally of significantly lower activity than wild-type, and yet two triple mutants containing E498D were found with improved activity. The triple mutant cycle of D469S/E498D/R520Q was therefore selected for a more detailed kinetic analysis.

**Fig. 3:**
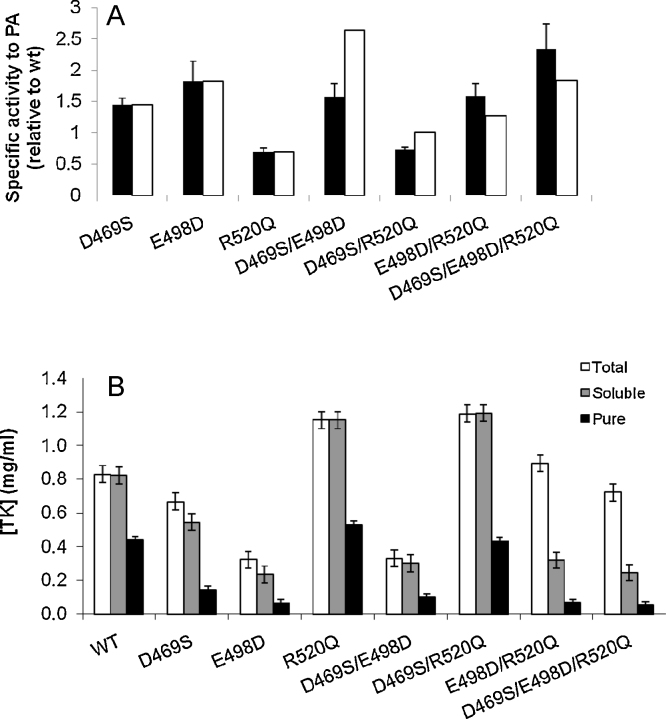
Specific activity and soluble expression of single, double and triple mutants of D469S, E498D and R520Q. (A) Specific activities towards propionaldehyde, relative to wild type for soluble enzyme in sonicated clarified lysates. (■) Experimentally determined. (□) Expected from the additive accumulation of improvements for the single mutants. (B) Impact of mutations on (□) total protein expression, () soluble fraction and (■) final purified enzyme concentrations.

A comparison of the lysate specific activities of the respective single, double and triple mutants of D469S/E498D/R520Q is shown in Fig. 3A, along with the specific activity expected for the accumulated independent effects of each single mutant. It is immediately clear that both D469S/E498D and D469S/R520Q exhibit negative synergy, where they have lower specific activity than that expected from the additive effects of each single mutation. Furthermore, E498D/R520Q and the triple mutant D469S/E498D/R520Q each exceed the additive effects of their respective single mutations, indicating positive synergies. The negative synergy on specific activity appears to be mostly linked to D469S, the only non-natural mutation, whereas positive synergy occurs between E498D/R520Q which also favours D469S when both mutations appear together.

The soluble protein concentrations obtained in the total lysate, soluble fraction and the final purified fractions were determined for each mutant (Fig. 3B) to identify any trade-off effects with specific activity. The total TK concentration in the cell lysate, relative to that of wild-type enzyme, was only marginally decreased for D469S, and 40% higher for R520Q, but is decreased to less than 40% for E498D, and in each case the majority of the protein remained in the soluble fraction. The codons used for mutagenesis were all the most optimal ones for expression in *E. coli* and so the decrease in expression for E498D is likely to be caused by a loss of protein stability or by protein mis-folding.

The total soluble protein for each single mutant was found to trade off against specific activity in the same way as for the previous single mutants (Fig. 1). However, the new double and triple mutants were also found to trade-off against soluble expression levels in the same way, indicating that they no longer precipitously lost both stability and function in the manner observed for the previous set of double mutants. Therefore, although all of the mutations that conferred an increase in specific activity were still destabilising to some degree, the synergy between the mutations at the three networked sites appeared to ensure that severe disruption of stability and function no longer occurred in the double or triple mutants.

R520Q generally appeared to decrease the specific activity and increase the soluble expression level of TK, except in E498D/R520Q and D469S/E498D/R520Q where it increased the total level of expression but not the amount of soluble enzyme. E498D containing mutants also all retained the same generally low level of soluble enzyme. Therefore, while the interaction of R520Q with E498D led to an improvement in specific activity, it also caused the enzyme to become more prone to aggregation into inclusion bodies. This suggests that the improvement of total expression achieved in all R520Q containing mutants, is not coupled to the destabilising effect of E498D that suppresses soluble expression. Instead, the increased expression conferred by R520Q appears to simply push the destabilised E498D towards aggregation. A similar effect was observed in the percentage of the total soluble protein that could be purified in each case using His-tag affinity resin, which remained the same in all mutants. However, for all variants containing the E498D mutation the protein was prone to aggregation after purification, giving total yields that were too low to carry out further analysis.

### 3.4 Kinetic analysis of purified transketolases

**Fig. 4:**
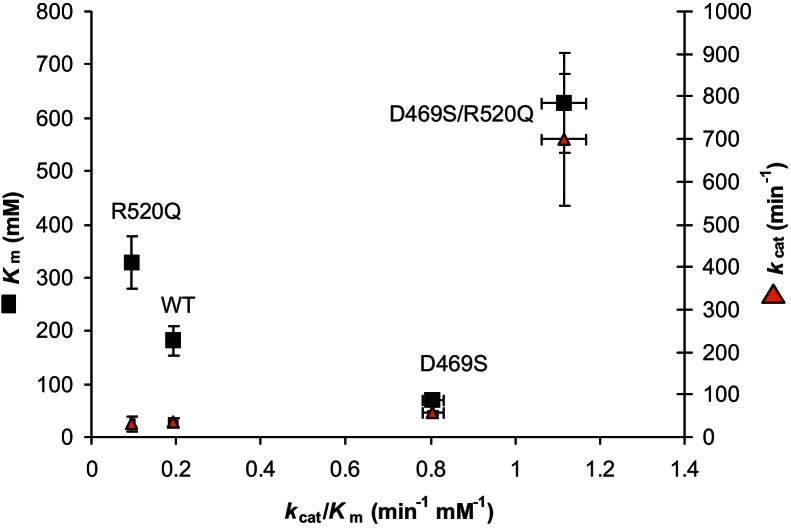
Comparison of *k*_cat_, propionaldehyde *K*_m_ and *k*_cat_/*K*_m_ for the double mutant cycle of D469S and R520Q, at 50 mM HPA, 50 mM Tris–HCl pH 7.0.

Enzyme kinetic parameters were determined for the purified D469S, R520Q and D469S/R520Q mutants as shown in Table 2 and Fig. 4. D469S was found to increase the *k*_cat_ by 60% and decrease the *K*_m_ of PA to 40% those of wild type, giving a 4-fold increase in the corresponding *k*_cat_/*K*_m_. R520Q had a similar *k*_cat_ but the *K*_m_ for PA was almost double that of wild type giving a 2-fold decrease in *k*_cat_/*K*_m_. By contrast, the double mutant D469S/R520Q yielded a significant 20-fold increase in *k*_cat_ compared to wild type, but a 3.5-fold increase in the *K*_m_ for PA. This demonstrates significant synergy between the D469S and R520Q mutations which mostly impacts on the *k*_cat_. The final specificity constant *k*_cat_/*K*_m_ was 5.8-fold greater than for wild-type.

The increase in *k*_cat_ to 700 min^−1^ mM^−1^ for D469S/R520Q is the highest obtained for any TK mutant to date. While the final *K*_m_ of 628 mM obtained for D469S/R520Q might appear to be poor in terms of substrate affinity, enzymes can actually be considered optimally efficient when the *K*_m_ is at least 10-fold greater than the substrate concentration, provided the *k*_cat_ is also increased accordingly (Fersht, 1974), or otherwise where *k*_cat_/*K*_m_ has reached the diffusion-controlled limit (Albery and Knowles, 1976). The increased *K*_m_ and the 20-fold increase in *k*_cat_ for D469S/R520Q is therefore a significant improvement of catalytic efficiency with *K*_m_ at 12.5× the 50 mM concentration of substrates used, and is also potentially useful for the biocatalytic synthesis of the chiral aliphatic keto-diol (3*S*)1,3-dihydroxypentan-2-one (DHP) at higher substrate concentrations.

### 3.5 Recombination of R520Q with previous D469 mutants

**Fig. 5:**
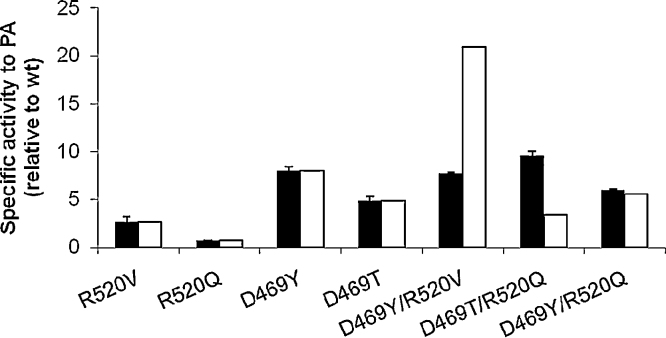
Effect of recombining R520Q or R520V with previously identified mutants D469T and D469Y upon their specific activities. (■) Experimentally determined with sonicated clarified lysates. (□) Expected from the additive accumulation of improvements for the single mutants.

To demonstrate that the D469T (or D469A) mutation in the previous double mutant libraries was not the common factor behind their critical loss of enzyme expression and function, given that D469S containing mutants did not have the same issue, we examined the additional mutants D469T/R520Q, D469Y/R520Q and D469Y/R520V. The D469T/R520Q mutant was observed to be more active than wild type in the initial double mutant screens (Table 1), but was not pursued further at that stage. D469Y was found previously to reverse the enantioselectivity of TK when using PA as the substrate, and also improved the specific activity. The latter two double mutants therefore allowed us to further test whether the increased expression in R520Q mutants might be generally beneficial to specific activities, relative to the R520V mutation previously identified to improve the overall activity of TK. Their specific activities are compared to those of the single mutants in Fig. 5.

D469Y/R520V showed negative synergy, whereas D469Y/R520Q showed no synergy. By contrast, D469T/R520Q showed positive synergy, fell on the upper envelope for the previous activity-expression trade-off curve (Fig. 1), and also resulted in the highest specific activity towards PA yet obtained for a TK mutant under these conditions. The improved activity for D469T/R520Q, compared to the loss of function in all previous recombinations with D469T, also showed that finding the right pairing of these residues is important, and that R520Q is able to provide positive synergy with some but not all D469 mutants. This is particularly interesting because the selection from libraries of double mutants containing R520Q would only happen if the initial D469S or D469T mutants were used as templates for R520 random mutagenesis, or if double mutant libraries of D469X/R520X were screened, as applied here, since R520Q on its own leads to partial loss of specific activity.

### 3.6 Enantioselectivity of transketolase variants

Mutants of TK have been found in our previous work that surprisingly both improved and reversed the enantioselectivity (Smith et al., 2008), particularly D469T and D469Y. The *e.e.s* of the DHP product obtained with the new mutants were determined and compared to previous results (Table 2). The stereoselectivity of wild-type TK for the *S*-enantiomer of the DHP product achieves a modest *e.e.* of 58%. Mutants containing D469S and D469T led to increases in *e.e.* to between 62%(*S*) and 68%(*S*). D469Y was known from previous work (Smith et al., 2008) to reverse the enantioselectivity to give an *e.e.* of 53%(*R*), and several repeat experiments here have revised this figure up to 68%(*R*). Adding R520Q to wild-type, D469T or D469Y left their *e.e.s* essentially unaltered at 58%(*S*), 68%(*S*) and 65%(*R*) in the respective double mutants, whereas adding R520V to D469Y improved the *e.e.* further to 85%(*R*), comparable with the previously obtained 88%*R* for H26Y (Smith et al., 2008).

## 4 Conclusions

Site-directed saturation mutagenesis targeted to individual active-site residues was previously found to be a useful strategy for identifying single mutants of transketolase that altered or improved activity, substrate specificity or enantioselectivity. Here we observed that the soluble expression levels for the single mutants appeared to trade off against specific activity gains, with a smooth distribution as expected from the introduction of mutations selected for activity but not stability (Bloom et al., 2006). However, recombining these previously identified activity-enhancing single mutants, to create double mutants, resulted in critical loss of protein expression and function.

The significant loss of function and soluble expression observed from just two mutations of this large (680 residue) enzyme was unexpected, and hinted at a strong synergy that could exist due to co-evolution between the targeted pairs of residues. Statistical coupling analysis of known transketolase sequences and those of closely related TPP-dependent enzymes confirmed that the beneficial single mutants identified previously were indeed at residues present in co-evolved networks. Therefore, the targeting of single functionally important sites with saturation mutagenesis, and selecting only for improved activity, appeared to identify single mutants with improved function but that also partially disrupted a network of coevolved residues such that they retained a just sufficient level of protein folding or stability. Their simple recombination therefore resulted in complete loss of function in nearly all of the double mutants, presenting a considerable potential barrier to directed evolution using this strategy. All but one double mutant disrupted a single nine residue network in the enzyme active site, and the remaining double mutant affected a separate network at the protein dimer interface.

Residue D469 was present at the heart of the active-site network, and yet several D469 mutants provided significant gains in activity towards PA, and also enantioselectivity, and so further evolution of these mutants was still desirable. To rectify this problem, four non-natural mutations of D469 that improve activity and enantioselectivity, were randomly recombined with just four naturally occurring variants at one or two neighbouring residues located within the same coevolved network. Screening of these libraries identified double mutants which minimised the loss of protein expression, and that synergistically improved *k*_cat_ and the specific activity towards propionaldehyde. The D469S/R520Q double mutant of TK obtained by this approach is not found in any of the natural 381 TPP-dependent enzyme sequences used to perform the SCA. For the natural sequences containing the R520Q mutation, the residue equivalent to D469 in TK is always co-mutated to a lysine in various related (phospho)pyruvate decarboxylases, or aspartate in several acetolactate synthases, but never serine. These findings indicate that natural sequences have not necessarily exploited all functional combinations of residues within coevolved networks. The existing combinations have been naturally selected for a specific function in the cell, so it is not necessarily expected that precisely the same natural combinations would be found when selecting for a new property such as altered substrate specificity. This approach therefore has significant potential to evolve an enzyme for synergistic improvements towards a non-natural property.

## Figures and Tables

**Table 1:** Libraries of TK mutants at sites in a single SCA cluster.

Residues mutated	Library of variants	Best variants from initial screen (activities^a^ > 70% wild type)
WT	–	–
G467	DVSA	**A**, **S**, D
D469	LTSA	**T**^b^, **L**, **A**^b^, S^b^
G470	TNLI	**T**
T472	SDLA	**S**, L
D495	NQEL	None
E498	DAVI	None
R520	GKQA	**A**, **G**^b^, **Q**
G467/D495	DVSA/NQEL	V/L, D/E
D469/R520	LTSA/GKQA	**T/Q**, **A/G**, **S/Q**, **S/G**
D469/E498	LTSA/DAVI	None
E498/R520	DAVI/GKQA	V/Q, D/K
D469/E498/R520	LTSA/DAVI/GKQA	**S/D/Q**, **S/D/A**, A/I/K, A/A/A, A/V/A

Libraries were limited to just four of the natural variants at each position commonly observed across the 17 enzyme types. Enzymes were assayed with 50 mM LiHPA, 50 mM propionaldehyde (PA), and 50 mM Tris–HCl, 2.4 mM TPP, 9 mM MgCl_2_, pH 7.0 to produce 1,3-dihydroxypentan-2-one (DHP).

a Activity (not specific activity) in decreasing order with those greater than wild type shown in bold.

b These mutants were also found in previous studies (Hibbert et al., 2007, 2008). All mutants were active, with at least 14% of wild-type activity.

**Table 2:** Kinetic parameters and enantioselectivities of TK mutants for the propionaldehyde (PA) substrate.

Enzyme	Specific activity (relative to wt)^a^	*k*_cat_ (min^−1^)	*K*_m_ (mM)	*k*_cat_/*K*_m_ (min^−1^ mM^−1^)	e.e. (%)
WT	1.0 ± 0.08	35 ± 10	181	0.19 ± 0.01	58 *S*
D469S	1.45 ± 0.1	57.1 ± 3.4	70.8	0.81 ± 0.02	62 *S*
E498D	1.8 ± 0.3				
R520Q	0.69 ± 0.06	31.4 ± 17	329	0.095 ± 0.005	58 *S*
D469S/R520Q	0.73 ± 0.03	700 ± 150	628	1.11 ± 0.05	67 *S*
D469S/E498D	1.6 ± 0.3				
E498D/R520Q	1.6 ± 0.16				
D469S/E498D/R520Q	2.3 ± 0.3				
D469T	4.9 ± 0.4				64 *S*
D469Y^b^	8.0 ± 0.5				68 *R*
R520V	2.6 ± 0.6				
D469T/R520Q	9.6 ± 0.5				68 *S*
D469Y/R520V	7.70 ± 0.05				85 *R*
D469Y/R520Q	5.99 ± 0.07				65 *R*

Specific activity of sonicates determined with 50 mM LiHPA, 50 mM PA, and 50 mM Tris–HCl, 2.4 mM TPP, 9 mM MgCl_2_, 250 mM NaCl, pH 7.0 to produce 1,3-dihydroxypentan-2-one (DHP). Standard deviations are given.

a Wild-type specific activity is 0.029 ± 0.001 μmol/mg/min (Hibbert et al., 2008).

b Repeated here and the final average incorporates the previously published raw data (Smith et al., 2008).
