# Supplementary material for: Directed evolution to re-adapt a co-evolved network within an enzyme
Source: J Biotechnol. 2012 Jan;157(1):237–45. doi: 10.1016/j.jbiotec.2011.11.017 (PMC3657141; doi:10.1016/j.jbiotec.2011.11.017)
Supplement: Supplementary file 1 [file mmc1.doc]

**Supplementary Information for:**

**Directed evolution to re-adapt a co-evolved network within an enzyme**

Corresponding author: E-mail: p.dalby@ucl.ac.uk

**Supplemental Data**

***Tables***

**Table S1. Mutagenic primers**

| Variant | (+) Primer sequence |
| --- | --- |
| A29E | GGTCACCCGGGG**GAA**CCTATGGGTATGGC |
| D259A | CGGTACCCAC**GCG**TCCCACGGTG |
| D259G | CGGTACCCAC**GGC**TCCCACGGTG |
| D259S | CGGTACCCAC**AGC**TCCCACGGTG |
| D259Y | CGGTACCCAC**TAT**TCCCACGGTG |
| H461S | GGTGATGGTTTACACC**AGC**GACTCCATCGGTCTGGG |
| D469Y | TCGGTCTGGGCGAA**TAT**GGGCCGACTCACCAG |
| R520V | GCACTGATCCTCTCC**GTG**CAGAACCTGGCGCAGC |
|  |  |
| G467D | ctccatcggtctg**gat**gaagacgggccgac |
| G467V | ctccatcggtctg**gtg**gaagacgggccgac |
| G467S | ctccatcggtctg**agc**gaagacgggcc |
| G467A | Ctccatcggtctg**gcg**gaagacgggccga |
| D469A | ggtctgggcgaa**gcg**gggccgactcacc |
| D469L | tccatcggtctgggcgaa**ctg**gggccgactcac |
| D469T | tccatcggtctgggcgaa**acc**gggccgact |
| D469S | tccatcggtctgggcgaa**agc**gggccgact |
| G470T | ggtctgggcgaagac**acc**ccgactcaccagccg |
| G470N | ggtctgggcgaagac**aac**ccgactcaccagccg |
| G470L | gtctgggcgaagac**ctg**ccgactcaccagc |
| G470I | ggtctgggcgaagac**att**ccgactcaccagccg |
| T472S | gaagacgggccg**agc**caccagccggttg |
| T472D | gcgaagacgggccg**gat**caccagccggttg |
| T472A | cgaagacgggccg**gcg**caccagccggttg |
| T472L | ggcgaagacgggccg**ctg**caccagccggttgag |
| D495E | tggcgtccgtgt**gaa**caggttgaatccgc |
| D495N | acatgtctacatggcgtccgtgt**aac**caggttgaat |
| D495Q | catggcgtccgtgt**cag**caggttgaatccgc |
| D495L | tacatggcgtccgtgt**ctg**caggttgaatccgcgg |
| E498I | tggcgtccgtgtgaccaggtt**att**tccgcggtcgc |
| E498D | ccgtgtgaccaggtt**gat**tccgcggtc |
| E498A | gtgtgaccaggtt**gcg**tccgcggtcgcgtg |
| E498V | gtgtgaccaggtt**gtg**tccgcggtcgcgtg |
| R520G | cactgatcctctcc**ggc**cagaacctggcgca |
| R520K | cgcactgatcctctcc**aaa**cagaacctggcgcagc |
| R520Q | cactgatcctctcc**cag**cagaacctggcgcag |
| R520A | gcactgatcctctcc**gcg**cagaacctggcgcag |

A1A2

B1

B2

**Figure S1.** Specific activities relative to wild-type for double mutants of single mutants previously identified as having improved specific activity towards glycolaldehyde (A1 & B1), and propionaldehyde (A2 & B2). A) Recombination of mutants previously found to improve specific activity on both GA and PA. B) Recombination of mutants previously found to improve specific activity on only PA. (■) Specific activities measured in sonicated clarified lysates. (□) Specific activities expected from the additive accumulation of improvements for the single mutants.


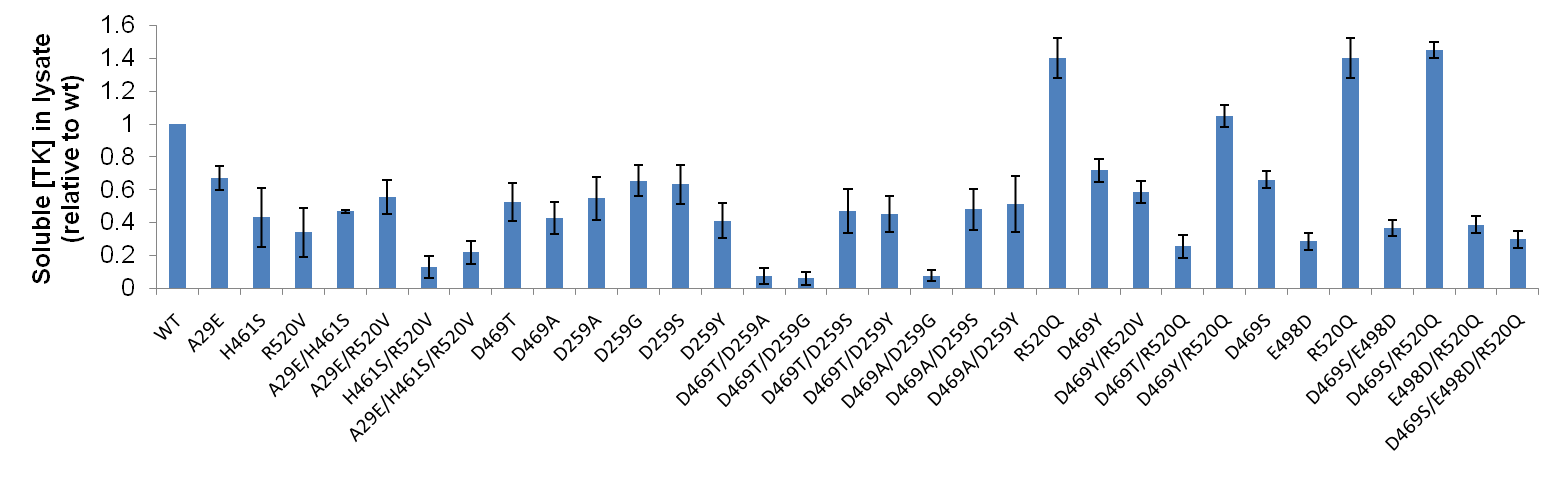


**Figure S2.** TK concentrations in each clarified lysate from sonication, relative to wild-type as determined by SDS-PAGE densitometry. WT concentration is 0.83 mg/ml.

**
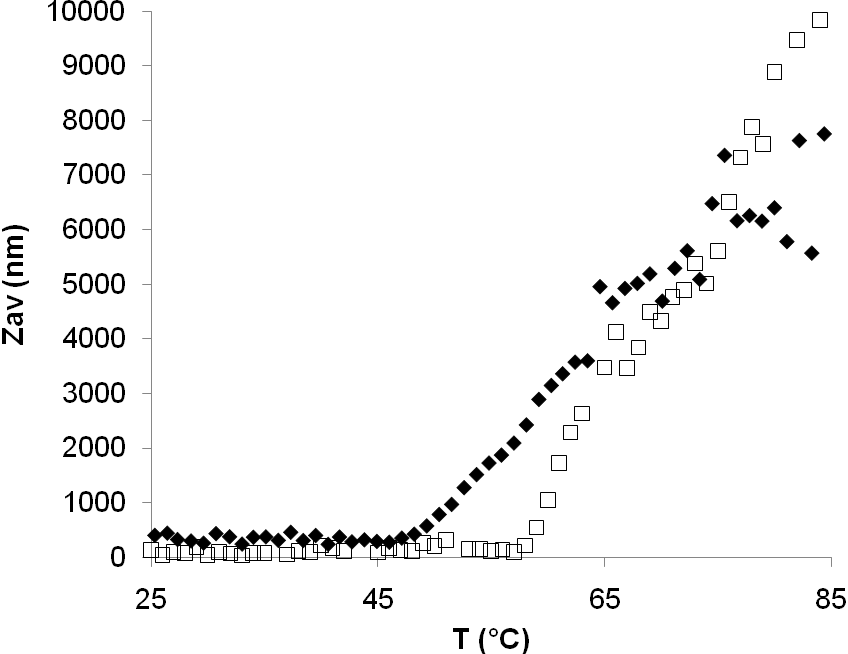
**

**Figure S3.** Temperature dependence of average particle size for (□) wild type, and (♦) D469T holo-TK determined by dynamic light scattering. TK at 0.1 mg mL^−1^ (1.38 μM) was incubated at 25 °C for 1 h in 25 mM Tris–HCl, pH 7.5, 5 mM MgCl_2_, 0.5 mM TPP prior to measurements. Temperature was increased at 1.0 °C per minute from 25 to 85 °C for each measurement of particle size distribution. Mean diameter (Zav) was calculated from the % intensity distribution at each temperature.

**Supplemental Experimental Procedures**

**Determination of aggregation temperature by dynamic light scattering**

The thermal denaturation of purified D469T transketolase was measured with a Zetasizer Nano S (Malvern Instruments Ltd., UK) as carried out previously for wild type (Jahromi et al., 2011). Holo-TK was prepared at 0.1 mg mL^−1^ (1.38 μM) in 25 mM Tris-HCl, pH 7.5, 0.5 mM TPP, 5 mM MgCl_2_ and incubated for 1 h prior to data acquisition. The temperature was increased at 1.0 °C per minute from 25 to 85 °C for each measurement of particle size distribution and controlled to ±0.1 °C using an integrated water bath unit. A control sample of buffers with cofactors was subtracted from each recording. Data were acquired in triplicate with a low volume disposable sizing cuvette with a path length of 1 cm. The average hydrodynamic diameters (Zav) of each sample were calculated from the % intensity measurements using the Zetasizer Nano Series software V.4.20 (Malvern Instruments Ltd., Worcestershire, UK), and then averaged.

### Statistical coupling energy calculations

SCA Matlab toolkit Version 1.5 (Suel et al., 2003) was used to calculate statistical coupling energies between sites in the multiple protein sequence alignments (MSA’s) of 382 TPP dependent enzymes comprised of residues 25-245 of the PP domain and residues 357-520 of the PYR domain. The MSA was converted using SCA version 1.5 into a matrix where each site is described by a 20 element vector of binomial probabilities of individual amino acid frequencies given their frequencies in all proteins (P_j_ = [P^ala^_j_, P^cys^_j_, P^asp^_j_,…, P^tyr^_j_]). P_j_^x^ gives the probability of the observed number of x amino acids at position j, given its mean frequency in all proteins. The 20 element vector of P_j_^x^ is then converted into a vector of statistical energies that represents the evolutionary constraint at site j (ΔG^stat^_j_ = [ΔG^ala^_j_, ΔG^cys^_j_, ΔG^asp^_j_,…, ΔG^tyr^_j_]). Each term in the vector is the value for amino acid x at site j and is given by ΔG^x^_j_ = kT* ln(P_j_^x^/P_MSA_^x^), where kT* is an arbitrary energy unit and P_MSA_^x^ represents a hypothetical site where all amino acids are observed at their mean frequencies in the MSA as a whole. This hypothetical site serves as a reference state for all sites and ΔG^x^_j_ represents the statistical free energy separating site j from the hypothetical site for amino acid x by the Boltzmann distribution. The magnitude of the ΔG^stat^_j_ vector represents an evolutionary conservation parameter for site j. To measure functional coupling between two positions j and i in the MSA, two statistical energy vectors were calculated, one from the full MSA (ΔG^stat^_j_) and one from a subalignment representing a perturbation of the amino acid frequencies at a second site i (ΔG^stat^_i|j_). The magnitude of the difference in these two statistical energy vectors represents a quantitative measure of the degree to which the probability of individual amino acids at site j is dependent on the perturbation at i, ΔΔG^stat^_j,I_ = ΔG^stat^_j_ - ΔG^stat^_i|j_. ΔΔG^stat^_j,i_ was calculated for all sites j given a perturbation at position i.

Calculation of evolutionary constraint at each site in the MSA revealed a diverse and well evolved collection of proteins. Figure S4a shows the evolutionary constraint at each position as measured by ∆G^stat^. Figure S4b and S4c display the frequencies of amino acid residues at an unconserved (position 38) and a highly conserved site (position 155) respectively, compared to the expected frequency of residues in all proteins. Neither site is expected to demonstrate significant coupling to other residues in the structure.

Figure S4. *a* Overall conservation across the MSA as measured by ΔG_stat_, an expression of divergence from the frequency of residues expected in all proteins. *b* Frequency of residues at position 38, the least conserved position in the MSA. The expected frequency of residues across all proteins is also shown. *c* Frequency of residues at the highly conserved position 155 versus the expected frequency of residues in all proteins.

### Validation criteria for alignments and acceptance of perturbations

In order for functional constraints in the MSA to be exposed, the alignment should have diversified to the extent that frequencies of amino acids at un-conserved sites relax near to their mean values in all natural proteins. The 5 least conserved sites in the MSA were identified with at least 85% occupancy (1QGD numbered positions: 38, 211, 363, 508, and 509), and the average ∆G^stat^ (Figure S5a) and the average ∆∆G^stat^ (Figure S5b) values were determined for these residues following the random elimination of increasing numbers of sequences from the MSA. The MSA was diverse enough such that random elimination of different numbers of sequences did not affect ∆G^stat^ at the least conserved sites (Figure S5a). Only subalignments of 80 or more sequences were allowed in the global SCA as those that resulted in less than 80 remaining sequences began to change the amino acid frequencies at the least conserved sites in the MSA, as measured by ΔG^stat^ (Figure S5a).

### Matrix assembly and cluster analysis

All acceptable perturbations, and their associated coupling energies to other positions in the sequence, are displayed as an initial matrix of statistical coupling energies (∆∆G^stat^) in Figure S6. Two-dimensional hierarchical clustering identified networks of co-evolved residues. Figure S7 shows the initial round of clustering. Following each round of clustering, large groups of perturbations or positions with low coupling energy were removed and the matrix was re-clustered to iteratively focus on the networks representing the highest statistical coupling. This process was repeated until the clusters converged and no further refinement was possible. After five rounds of iterative clustering we identified a highly coupled network of 30 residues and 45 specific perturbations (Figure S8).

Figure S5. The 5 least conserved sites that retain at least 85% occupancy were selected. *a* Average dGstat at these 5 sites was evaluated following random elimination of increase in proportions of the MSA. *b* Average ddGstat values at the 5 sites following random elimination.

**Figure S6**. Initial matrix of statistical coupling energy (∆∆G^stat^) values following global statistical coupling analysis of the combined PP and Pyr domains of TPP dependent enzymes. Columns represent specific perturbations of the multiple sequence alignment. Rows represent positions in the PP and PYR domains. ∆∆G^stat^ values between residues are displayed as a linear colour scale from least interaction, blue (0) to the strongest interactions, deep red (4).

Figure S7. Matrix obtained after the initial round of 2-dimensional clustering.

Figure S8. Final global SCA matrix following iterative focusing and reclustering around areas of high signal. As in previous figures, perturbations are represented by columns and positions by rows. ∆∆G^stat^ values between residues are displayed as a linear colour scale from least interaction, blue (0) to the strongest interactions, deep red (4). Dendrograms indicate networks of coupled residues which cluster according to their responses to perturbations. Final matrix and dendrogram of the combined PP-Pyr-domains revealed the six interconnected clusters shown in different colours on the right.

### Single PYR domain global SCA analysis

The MSA was divided and global SCA carried out as described above for the PYR domain alone. Initial clustering resulted in the matrix and dendrograms shown in Figure S9a. Iterative clustering then focussed this down onto an interesting self-selecting cluster containing the D469 and R520 residues known to be involved in the active site, to give the final matrix in Figure S9b. The higher signal clusters in Figure S9a again contained residues involved in intersubunit interactions.

**Figure S9.** Hierarchical clustering of statistical coupling energies for the Pyr domains of TPP-dependent enzymes. Columns represent specific perturbations of the multiple sequence alignment. Rows represent positions in the protein. ∆∆G^stat^ values are displayed as a linear colour scale from least interaction, blue to the strongest interactions, deep red. Dendrograms indicate networks of co-evolved residues. **a)** Matrix and dendrogram for the initial clustering of only Pyr-domain residues. The cluster shown in red on the dendrogram contains two key active-site residues D469 and R520. **b)** Final matrix and dendrogram for the Pyr-domain following iterative focusing and re-clustering.

**Supplemental References**

Jahromi, R. R. F., Morris, P., Martinez-Torres, R. J. and Dalby, P. A. (2011). Structural stability of *E. coli* transketolase to temperature and pH denaturation. J. Biotechnol. 155, 209-216.

Suel, G. M., Lockless, S. W., Wall, M. A., Ranganathan, R., 2003. Evolutionarily conserved networks of residues mediate allosteric communication in proteins. Nat. Struct. Biol. 10, 59-69.
